# An assessment of knowledge, attitude and practices (KAP) towards diabetes and diabetic retinopathy in a suburban town of Karachi

**DOI:** 10.12669/pjms.311.6317

**Published:** 2015

**Authors:** Muhammad Saleh Memon, Sikander Ali Shaikh, Abdul Rashid Shaikh, Muhammad Faisal Fahim, Seema N. Mumtaz, Nadeem Ahmed

**Affiliations:** 1 Dr. Muhammad Saleh Memon, DO, FRCS (Eden), Director Projects, Isra Ophthalmic Research & Development Center,; 2 Sikander Ali Shaikh, M.A (Sociology), Project Manager, Department of Community Based Projects,; 3 Dr. Abdul Rashid Shaikh, MCPS, MS, Assistant Professor, Department of Ophthalmology,; 4 Muhammad Faisal Fahim, M.Sc (Statistics), Statistician, Isra Ophthalmic Research & Development Center; 5 Dr. Seema N. Mumtaz, MBBS,M.Phill,MPH,MBA(Health), DCPS(HSCM), Chairperson, Community Medicine, Department of Community Medicine, Al Tibri Medical College, Isra University Karachi Campus, Pakistan.; 6 Mr. Nadeem Ahmed, M.A Econ, MES Environmental Studies, Consultant, Isra Ophthalmic Research & Development Center,

**Keywords:** Diabetic retinopathy (DR), Diabetic Education Program (DEP), Knowledge, Attitude and Practices (KAP)

## Abstract

**Objective:**

To assess the Knowledge, Attitude and Practices (KAP) towards diabetes and diabetic retinopathy in the general population of Bin Qasim Town (BQ), Karachi.

**Methods:**

An observational, cross-sectional study was approved by Research Ethical Committee of Al-Ibrahim Eye Hospital. It included every third household by stratified sampling in each Union Council of (BQ) Town, in the months of May to July 2013. The interview Questionnaire included 43 questions, of qualitative and quantitative aspects, which were awarded 56 scoring points. SPSS version 20.0 was used to analyze the data.

**Results:**

Six hundred ninety two adults one from each household were interviewed. Of the total respondents, 271 (39.2%) had diabetes. Lowest mean knowledge score (5.28 ± 6.09) was seen in illiterate respondents. Male’s Mean Knowledge score (7.61 ± 6.600) was better than female’s (5.46 ± 6.21) with P <0.001. Over all mean score of Attitudes towards diabetes was 5.43 ± 2.57. It was higher (6.62 ± 2.03) in diabetic respondents as compared with non-diabetic respondents (4.70 ± 2.59) with p < 0.000. In Practice module majority of the respondents (69.9%) did not exercise, 49% took high caloric snacks between meals and 87% ate outside home once a month, 56.8% diabetics visited ophthalmologist for routine eye examination; but only 9.2% asked for retinal examination.

**Conclusion:**

Lack of knowledge of diabetes was found in the surveyed community, more marked in females, illiterate and the individuals not having diabetes.

## INTRODUCTION

Diabetic Retinopathy (DR) is a well-recognized complication of diabetes mellitus. Out of 39 million global blindness due to various eye diseases, 4.8% (1.8 million) is due to Diabetic Retinopathy (DR)^1^^-^^3^ Nationally every fourth patient with diabetes has some level of DR^4^^,^^5^ with improved care the diabetics are living longer and are exposed to the risk of chronic complications like DR resulting in increasing blindness. Health care providers are exploring ways and means to control blindness due to diabetes. Timely treatment of diabetes and regular screening for complications can reduce or delay the complications of diabetes by as much as 50%.^6^ This needs highly trained Human resource and costly sophisticated equipment. In low economy countries Prevention of diabetes through awareness and education of the community is the most cost effective management of diabetes and its related complications.^7^^,^^8^ In order to create awareness in the community, insight into the gaps of knowledge, attitudes and practices regarding diabetes and blindness due to diabetes is important. Base line study was done in 2012^9^ in one of the rural town of Karachi^. ^In order to enhance the information, a study was conducted in Bin Qasim town which is a semi urban area that comprises of mixed population in respect of ethnicity, occupation and education.

Bin Qasim (BQ) Town is geographically located in eastern part of Karachi and has population of 315,684 (1998 census). Administratively it is divided into 7 union councils. The incidence of poverty and illiteracy are high resulting in poor eye care behavior.

This town has 13 public sector health facilities, 09 lady health supervisors (LHS) and 247 lady health workers (LHW).There is one secondary health care center at Union Council Ibrahim Hyderi, one rural health center at Rehri, three Basic Health Unit one each in UC 7, UC 3 (cattle colony) and UC 5 (Landhi), 34 dispensaries and one Maternity Child Health centers in UC 7. There is one tertiary level private hospital in UC Gulshan-e-Hadeed and a tertiary eye hospital run by an NGO adjacent to this town.

Aim of this study was to assess the knowledge, attitude and practice of the people in Bin Qasim Town, Karachi, regarding diabetes and DR.

## METHODS

The study design was qualitative with a mix of descriptive, cross sectional and exploratory research design tools. The descriptive design allows researcher to obtain information about the current status of the phenomenon while exploratory design familiarizes the researcher with basic details, settings and insights about the problem that have not been studied so far.

A pre-tested questionnaire was developed to investigate community behavior towards key research questions. The questionnaire is based on both quantitative and qualitative research variables that form basis for use of mix method approach for in-depth contextualization of research question.

Before starting study, the permission from Research Ethical Committee (REC) of Al-Ibrahim Eye Hospital was obtained. Informed consent was obtained from individual respondents and community leaders. Ethical considerations were fulfilled by obtaining verbal consent and maintaining the confidentiality. The primary data collection tool was field survey of the household in the selected sample area, for key demographic indicators secondary sources of information were also utilized.

The primary sampling unit (PSU) was union council in which sample population was drawn through random sampling technique. The study includes all seven union councils of Bin Qasim Town namely Ibrahim Hyderi, Rehri, Cattle Colony, Quaidabad, Landhi, Gulshan-e-Hadeed and Ghaghar Phatak. Sample selection in union council was done through stratified sampling procedure and 100 questionnaires were conducted in each UC. The response rate for the study sample was 98.8% (692/700*100). Total sample of the study was 692 households. One respondent (male or female) from each sample household was selected from all clusters in UCs. Sampling technique was a non-probability quota sampling.

***Questionnaire Design and Data Analysis: ***The idea of this questionnaire was obtained originally by a study Asad Jiskani et al.^9^ with few modifications. The questionnaire was designed to capture five important aspects of KAP among the population in the sample area. This includes respondents’ attitude, knowledge about diabetes and related complications, risk factors, treatment of diabetes, monitoring of diabetes and usual practices in daily life.

To measure the levels of various aspects of Knowledge, Attitude and Practice (KAP), the questionnaire was divided into three distinct modules. In each module, relevant questions were asked from the respondents such as in Knowledge module the emphasis was given to assess the level of knowledge of respondents for diabetes and Diabetic Retinopathy. To assess knowledge, attitude and practices, 17, 10 and 16 questions were asked respectively. 

The analysis of three modules was done on the basis of scalar-scoring method. There were two types of questions. Those questions having two possible answers were given 1 point for correct response and zero point for wrong or uncertain response. The other type of questions had 3 levels of scores, 0, 1, & 2 representing Poor, Fair and Good level of Knowledge, Attitude or Practice. Total KAP score is used to rank the level of knowledge, attitude and practice and subsequent qualitative analysis was conducted to rank high, medium and low scores. 

Overall, there were 43 questions in the questionnaire. If a person answered all questions correctly, 56 scoring points were awarded. The total 56 points were divided into three sections in which 23 points (41%) attributed to knowledge section, 10 points (18%) to attitude and 23 points (41%) to practices. Those respondents who obtained KAP score above 50 were considered as high level, while the scores between 25 and 50 were considered as medium level. The score below 25 was considered as low level.

All the categorical variables were presented as frequencies and percentages and all the continuous variables were shown as Mean ± Standard Deviation. To compare KAP scores, One Sample Independent T-test and One Way ANOVA was used to know the level of significance of variables. P –value < 0.05 considered statistically significant.

## RESULTS

Socio-economic Profile of Household: The response rate for the study sample was 98.8% (692/700*100). Total sample size of this study was 692 households with 5216 total number of family members, with an average household size of 7.6 members. Out of total 692 respondents, 248 (35.8%) were male and 444 (64.2%) females. Mean age of the respondents was 46.85 ±12.75 years, with minimum age of 20 years and maximum age of 90 years. Table-I shows the socio-economic profile of the respondents.

Knowledge of Diabetes: Out of 692 respondents 333 (48.12%) were totally unaware about diabetes. The remaining 359 (51.88%) respondents who claimed to have knowledge of diabetes were asked about the symptoms, causes, complications of diabetes and its possible impact on eyes. About 29.8% of 359 respondents failed to show any knowledge of various aspects of diabetes. Remaining 70% had some knowledge about clinical (49.3%) as well as scientific (20.9%) aspects.

Table-II shows the respondents claiming to have Knowledge regarding Diabetes (n=359). According to overall scoring of knowledge section, 60% of the respondents had poor/no knowledge regarding various aspects of diabetes mellitus, 40% of respondents had fair knowledge while only (16.9%) had good knowledge.

Attitude towards diabetes: Out of 692 respondents, 431 (62.3%) believed that diabetes and its complications can be prevented but 55.9% had either poor or no knowledge of various strategies to do this.

Responding to the question regarding association of dietary pattern and diabetes, 69.2% of the respondents believed that routine dietary pattern did not predispose to diabetes. Importance of exercise in prevention and control of diabetes was recognized by 406 (58.7%) and role of frequent blood sugar tests was realized by 592 (85.5%) of the respondents. Role of eye examination by an ophthalmologist in prevention and control of Diabetes related Blindness was recognized by 58.8%; but only 25.6% of the respondents go for detailed retina examination by an ophthalmologist.

Overall mean score of attitudes towards diabetes was found to be 5.43 ± 2.57. It was higher (6.62 ± 2.03) in diabetics as compared to non diabetic (4.70 ± 2.59) with significant statistical difference (P-value0.000) and higher in males (5.86 ± 2.46) than females ((5.19 ± 2.61) with significant statistical difference (P-value 0.001). 

**Fig.1:**
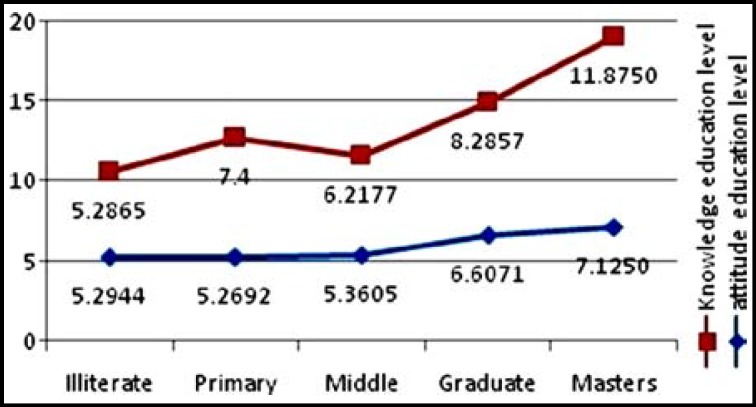
Association of mean knowledge / attitude score with levels of education.

**Fig.2:**
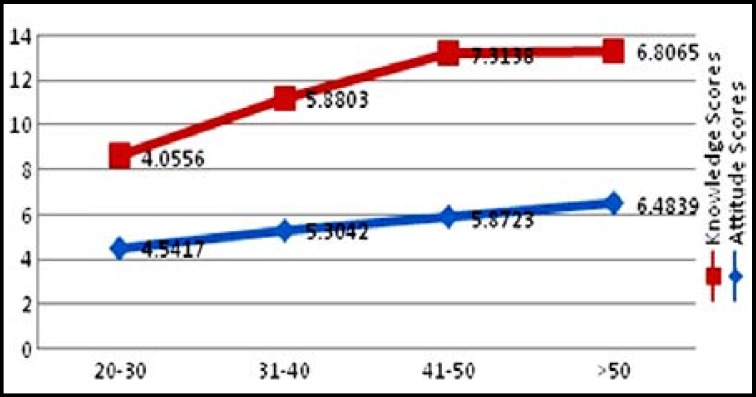
Mean knowledge / attitude scores with respect to age group.

The linear relationship of level of education, knowledge and attitude of the respondents is shown in (Fig.1). Similar relationship exists between knowledge, attitude and age of the respondents. (Fig.2).

Practices: Various pertinent questions were asked regarding risk, prevention, management of diabetes and Diabetes related Blindness. The responses are mentioned in Table-III.

## DISCUSSION

There exists enough evidence to show that not only risk of diabetes can be reduced by life style change,^6^ but the risk of Diabetic Retinopathy (DR) to sight can also be greatly reduced by good blood glucose and blood pressure control, effective screening and laser treatment.^10^^,^^11^ Effective screening and laser will need good infra structure, highly trained human resource, established referral chain and willingness of the diabetic patients to avail the available facility. It is the dilemma of the developing countries that the service- intake by the community is not more than 30%^12^ mainly because of the unawareness of the community regarding chronic complications of diabetes and its early management.

**Table-I:** Socio Economic Status.

**Gender of Respondent**	**Frequency (%)**
Male	248 (35.8%)
Female	444 (64.2%)
Total	692
Diabetic respondents	271 (39.2%)
Non -diabetic respondents	421 (60.8%)
Age of Respondent	
20-30	72 (10.4%)
31-40	401 (57.9%)
41-50	188 (27.2%)
>50	31 (4.5%)
Total	692
Education of Respondent	
Illiterate	377 (54.5%)
Primary	104 (15.0%)
Middle	147 (21.2%)
Graduate	56 (8.1%)
Masters	8 (1.2%)
Total	692
Profession of Respondent	
Unskilled labor	286 41.3%)
House Wife	153 (22.1%)
Skilled Labor	77 (11.1%)
Government Service	74 (10.7%)
Private service	64 (9.2%)
Unemployed	22 (3.2%)
Retired	16 (2.3%)
Total	692

Date shown in frequency and percentages n (%)

**Table-II:** Answers of the respondents claiming to have knowledge regarding diabetes (n=359).

**Questions regarding Knowledge of Diabetes**	**No knowledge**	**Fair knowledge**	**Good knowledge**
What are the symptoms of diabetes?	37 (10.3)	79 (22)	243 (67.7)
What are the causes of diabetes?	115 (32.0)	157(43.7)	87 (24.2)
What complications diabetes can cause?	13(3.6)	125(34.8)	221 (61.6)
What effects does diabetes have on eyes?	155(43.2)	170(47.4)	34 (9.5)
What is the treatment of diabetes?	54 (15)	277(77.2)	28 (7.8)
How diabetes can be prevented?	110 (30.6)	235(65.5)	14 (3.9)
What foods, if taken frequently, can increase the risk of diabetes?	59 (16.4)	6 (1.7)	294 (81.9)
How can diabetes related eye complications be treated?	108 (30.1)	239 (66.6)	12 (3.3)

**Table-III:** Showing practices of the respondents

**Practice**	**Percentage**
High Calories between meal	49%
Eating out once a month	87%
No exercise	67.9%
Regular blood sugar testing	87.5% in diabetics and 9.3% in non diabetics
Other relevant investigations like HbA1c,lipid profile	22.1%
Professional seeking habit	77.9%
Visit ophthalmologist	56.8%
Ask for retinal screening	9.2%

Gaddap study^9^ had shown that over all knowledge of the sample population was 35.2% and only 9.5% of the respondents were aware of any risk factors of diabetes and diabetic retinopathy.

BQ study has shown almost similar results. With half of the population (54.5%) illiterate, majority of the respondents (48.2%) did not have any knowledge of diabetes. Most of the people 56.6% did not know if diabetes was related to diet, and 63.7% did not know the impact of disease on the eyes. Poor knowledge was reflected in the attitude of the community as most of the people (69.2%) did not believe that their routine dietary pattern was a risk factor regarding diabetes and Diabetic retinopathy. However few positive attitudes emerged from the study. Importance of exercise in prevention and control of diabetes was recognized by 58.7%, role of frequent blood sugar tests was realized by 85.5% and role of eye examination by an ophthalmologist in prevention and control of diabetic retinopathy was recognized by 58.8%.

Practices of the community follow the knowledge and attitude pattern. Strengths noted were frequent blood sugar checking (87.5%), seeking professional help for control of disease in 77.9% and visit to ophthalmologist found in 56.8%. Weaknesses included taking high caloric snacks between meals (49%), eating outside home at least once a month (87%) , poor attention to other blood tests like lipid, glycated haemoglobin HbA1C (22.1%) and retinal screening in 9.2% of the respondents. There existed discrepancy between the belief and practice regarding exercise. Importance of exercise was recognized by 58.7%; but 67.9% did not do any exercise. People believe that physical exercise is good thing but cannot do it for many reasons, which are to be explored. The study has shown that diabetics do visit the physicians and ophthalmologists. Responsibility lies on the shoulders of the family physicians and ophthalmologists to educate the patient regarding control of diabetes and its chronic complications. There exists clinical evidence that increasing awareness in the community regarding management of diabetes is an effective method of controlling chronic complications due to diabetes. In many countries Diabetic education program (DEP) has proved cost effective preventive strategy.^13^^-^^15^ The results of Gaddap town and BQ towns can make basis of DEP in Pakistan by developing “Behavioral Change Communication Material” to initiate DEP in two towns of Karachi.

## CONCLUSION

Lack of knowledge of diabetes was found in the surveyed community, more marked in females, illiterate and the individuals not having diabetes. Poor knowledge and practices regarding diabetes and diabetes related blindness in the community are important weaknesses to be addressed. Physician seeking habit, regular blood checking habit and visit to ophthalmologists were strengths to be used by the family physicians and ophthalmologists to educate the patients.

***Suggestion: ***Behavioral Change Communication material for Awareness & Education has to be pictorial and method of dissemination has to be audio visual in addition to print media. Females, illiterate and younger age groups need more attention in any campaign of awareness and education. Role of Family physicians in responding properly to the diabetics by creating awareness in the community regarding diabetes and its complication is very crucial. Referral of patients with diabetes to ophthalmologist for early screening is important. Ophthalmologists should screen every diabetic for Diabetic retinopathy even if not asked. Advocacy of family physicians and ophthalmologists is an integral component of DEP.
